# N–O–S Co–Doped Hierarchical Porous Carbons Prepared by Mild KOH Activation of Ammonium Lignosulfonate for High–Performance Supercapacitors

**DOI:** 10.3390/nano15211633

**Published:** 2025-10-26

**Authors:** Zhendong Jiang, Xiaoxiao Xue, Yaojie Zhang, Chuanxiang Zhang, Wenshu Li, Chaoyi Jia, Junwei Tian

**Affiliations:** 1 Department of Mining Engineering, Shanxi Institute of Technology, Yangquan 045000, China; 18623855902@163.com (Z.J.); 19722743666@163.com (W.L.); 18035324889@163.com (C.J.); 13033479307@163.com (J.T.); 2 Mingde College, Henan University of Technology, Zhengzhou 450001, China; 3 College of Chemical Engineering, Ordos Institute of Technology, Ordos 017000, China; 4 Ordos Institute of Clean Coal Development and Utilization, Henan Polytechnic University, Ordos 017000, China; zcx223@163.com

**Keywords:** ammonium lignosulfonate, N–O–S co-doped, hierarchical porous carbon, supercapacitors

## Abstract

The development of porous carbon materials that meet the demands of commercial supercapacitors is challenging, primarily due to the requirements for high energy and power density, as well as large-scale manufacturing capabilities. Herein, we present a sustainable and cost-effective method for synthesizing N–O–S co-doped hierarchical porous carbons (designated as ALK_x_) from ammonium lignosulfonate (AL), an industrial by–product. This process employs a low KOH/AL mass ratio (x ≤ 0.75) and a carbonization temperature of 900 °C. The resulting materials, ALK_0_._50_ and ALK_0.75_, exhibit an exceptionally high specific surface area (>2000 m^2^ g^−1^), a well-balanced micro-mesoporous structure, and tunable heteroatom content, which collectively enhance their electrochemical performance in both aqueous and ionic liquid electrolytes. Notably, ALK_0.75_ features a heteroatom content of 13.2 at.% and a specific surface area of 2406 m^2^ g^−1^, owing to its abundant small mesopores. When tested as an electrode in a two–electrode supercapacitor utilizing a 6 M KOH electrolyte, it achieves a high specific capacitance of 250 F g^−1^ at a current density of 0.25 A g^−1^ and retains 197 F g^−1^ even at 50 A g^−1^, demonstrating remarkable rate capability. In contrast, ALK_0.50_, characterized by a lower heteroatom content and an optimized pore structure, exhibits superior compatibility with the ionic liquid electrolyte EMIMBF_4_. A symmetric supercapacitor constructed with ALK_0.50_ electrodes attains a high energy density of 90.2 Wh kg^−1^ at a power density of 885.5 W kg^−1^ (discharge time of 60 s). These findings provide valuable insights into heteroatom doping and the targeted regulation of pore structures in carbon materials, while also highlighting new opportunities for the high-value utilization of AL.

## 1. Introduction

Supercapacitors play a vital role in the new energy sector as electrochemical energy storage components. They have attracted significant attention from researchers due to their numerous advantages, including high capacity density, rapid charging and discharging rates, extended cycle life, wide operating temperature range, and high safety [1,2,3,4,5,6]. Supercapacitors can be classified into two fundamental categories based on their charge storage mechanisms. Double-layer capacitors (EDLCs) store energy through interfacial electrostatic forces, enabling the physical adsorption and storage of electric charges. In contrast, pseudocapacitors store energy through reversible Faradaic reactions occurring on the surface of the electrode active materials [7,8,9]. This mechanistic distinction directly influences their performance metrics. EDLCs typically exhibit higher power density and superior cycling stability, while pseudocapacitors generally offer higher energy density.

Porous carbon materials are primarily used in supercapacitor electrodes due to their high specific surface area for charge accumulation, inherent electrical conductivity for efficient charge transport, and cost–effectiveness for large-scale production [10,11,12]. Despite their superior power output and exceptional cycle life, the broader commercial use of carbon-based supercapacitors in energy storage systems is limited by their relatively lower energy density compared to modern battery technologies [13,14,15]. To bridge this performance gap, current research focuses on increasing the energy density of supercapacitors while maintaining their high power delivery and cycling stability. This effort mainly targets two areas: developing advanced electrode materials with optimized porous structures and formulating novel electrolyte systems to expand the operational voltage window [16,17]. The energy storage capacity (E) of a supercapacitor is fundamentally determined by the equation, where increasing the operational voltage (V) and maximizing the specific capacitance (C) of the electrode are the primary methods to achieve higher energy density [18,19]. Expanding the voltage window, often achieved through non-aqueous electrolytes like ionic liquids, provides a quadratic increase in energy density [20]. Simultaneously, developing advanced electrode materials with high specific capacitance directly enhances the charge storage capacity per unit mass. Therefore, the strategic co-optimization of these interdependent parameters is crucial for designing next-generation supercapacitors with competitive energy performance.

The operational voltage range of a supercapacitor is primarily determined by the electrolyte’s electrochemical stability. Aqueous electrolytes, known for their smaller hydrated ion radii and superior ionic conductivity, can enhance specific capacitance [21,22,23]. In contrast, non–aqueous systems, such as ionic liquid electrolytes, offer a wider electrochemical stability window (typically around 4 V, compared to approximately 1.23 V for aqueous systems), allowing commercial supercapacitors to operate at higher voltages and significantly increase energy density [24,25,26]. The specific capacitance of carbon–based supercapacitors is affected by a complex interplay of factors beyond just specific surface area. Pore architecture, particularly the hierarchical distribution of micropores (50 nm), is crucial for ion transport kinetics and electrolyte accessibility [27,28,29,30]. An optimal balance is achieved when micropores provide extensive surface area for high charge storage, mesopores function as low–resistance channels for efficient ion diffusion to interior surfaces, and macropores act as ion-buffering reservoirs that minimize diffusion distances, especially at high current densities. Additionally, surface functionalization through heteroatom doping (e.g., with oxygen or nitrogen) can introduce pseudocapacitive effects via reversible faradaic reactions. These functional groups enhance electrode wettability, facilitate ion adsorption, and improve charge storage capacity without significantly affecting cycling stability. Integrating these interconnected pore sizes into a hierarchical architecture is a crucial design strategy for advanced carbon–based supercapacitor electrodes. This multi–scale porous network enhances potential capacitance and improves rate capability and cycle stability through synergistic effects [31,32,33,34,35].

Incorporating heteroatoms like N, O, and S into carbon-based electrode materials is crucial for enhancing supercapacitor performance in aqueous electrolytes. These heteroatoms significantly alter the carbon matrix’s intrinsic properties, leading to improvements through two main, often synergistic, mechanisms [36,37,38,39,40,41,42,43]. Although the KOH activation method is commonly used to synthesize commercial porous carbons, the typically high dosage of the activator raises safety concerns and complicates purification [44]. Thus, selecting the right precursor is essential, as it fundamentally determines the final material’s characteristics—such as porosity and surface chemistry—especially when aiming for high performance with minimal KOH usage [45]. Lignosulfonate, abundant in aromatic structures and heteroatoms, is readily available through various pulping processes and holds promising potential for porous carbon preparation [46]. We present a simple and cost–effective method for synthesizing N–O–S co-doped hierarchical porous carbons, designated as ALK_0.50_ and ALK_0.75_. This involves a gentle KOH activation process on AL with KOH/AL mass ratios below 0.8. When the KOH/AL ratio reaches or exceeds 0.5, the activation process significantly enhances the formation of small mesopores (2–4.5 nm) within the carbon framework. The ALK_0.75_ material, with its well-developed mesoporosity and N–O–S triple doping, exhibits outstanding electrochemical performance in a 6 M KOH aqueous electrolyte. In a two–electrode system, it achieves a high specific capacitance of 250 F g^−1^ at 0.25 A g^−1^ and maintains an impressive rate capability of 197 F g^−1^ even at 50 A g^−1^. Conversely, the ALK_0.50_ sample, with improved electrolyte wettability, shows exceptional energy storage properties in the ionic liquid EMIMBF_4_. A symmetric supercapacitor using ALK_0.50_ delivers notable energy and power densities of 90.2 Wh kg^−1^ and 885.5 W kg^−1^, respectively, and retains an energy density of 34.2 Wh kg^−1^ at a high power density of 14.1 kW kg^−1^.

## 2. Experimental Section

### 2.1. Materials

All chemical reagents were used as received without additional purification. The main materials were AL with a nominal purity of approximately 65% (It’s content of each component is shown in Table S1), sourced from Shanghai Tingruo Chemical Co., Ltd. (Shanghai, China), and potassium hydroxide (KOH, ACS reagent grade) from Shanghai Chemical Co., Ltd. (Shanghai, China). The ionic liquid electrolyte, 1-ethyl-3-methylimidazolium tetrafluoroborate (EMIMBF_4_), was acquired from Lanzhou Greenchem ILs (Lanzhou, China).

### 2.2. Preparation of ALK_x_

**Figure 1:**
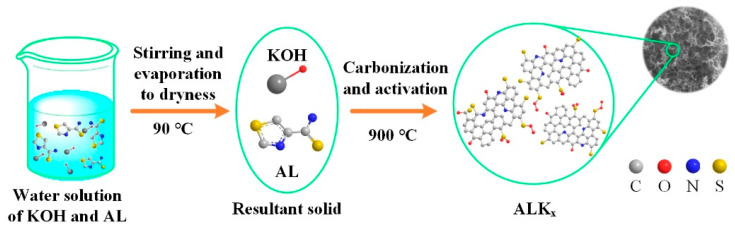
Schematic illustration of the production processes for the ALK_x_.

The synthesis procedure for the ALK_x_ materials is outlined in Figure 1. In a typical process, AL and KOH were mixed in deionized water at specified mass ratios (KOH/AL = 0.25:1, 0.50:1, and 0.75:1) under magnetic stirring until complete dissolution. The homogeneous mixture was then maintained at 90 °C with continuous agitation to evaporate the water. The solid precursor was carbonized in a tube furnace at 900 °C for 1 h under a continuous N_2_ atmosphere, with the temperature ramping at a controlled rate of 5 °C·min^−1^. Following carbonization, the resulting black residues underwent successive washing cycles with deionized water until a neutral pH was achieved and were subsequently dried at 100 °C for 4 h. This process yielded a series of porous carbons, labeled as ALK_x_ (where x = 0.25, 0.5, 0.75), with the subscript denoting the mass ratio of KOH activator to precursor used in the preparation. To evaluate the specific role of KOH in pore development, a control sample (ALK_0_) was synthesized in parallel using the exact same procedure but in the absence of KOH.

### 2.3. Characterizations

Morphological characteristics of the ALK_0_ and ALK_x_ samples were observed using scanning electron microscopy (SEM, LEO1530, LEO Electron Microscopy, Oberkochen, Germany). Their microstructural details were characterized with transmission electron microscopy (TEM, TecnaiG20, 200 kV, Thermo Fisher Scientific, Hillsboro, OR, USA). Nitrogen adsorption–desorption isotherms were measured at 77 K on a Quantachrome AutosorbiQ–MP system (Quantachrome Instruments, Boynton Beach, FL, USA). Specific surface area was calculated using the Brunauer–Emmett–Teller (BET) model. Total pore volume was derived from the nitrogen adsorption capacity at a relative pressure (P/P_0_) of 0.99. Pore size distributions were acquired via the Density Functional Theory (DFT) approach. Crystalline structure and phase composition were analyzed by X-ray diffraction (XRD, SmartLab 9kw, Rigaku Corporation, Tokyo, Japan) with Cu Kα radiation (λ = 1.54056 Å). Carbon structure was further probed using Raman spectroscopy (LabRam HR800, HORIBA Jobin Yvon, Loos, France). Surface chemical composition was determined through X-ray photoelectron spectroscopy (XPS, Thermo Escalab 250XI, Thermo Fisher Scientific, Waltham, MA, USA).

### 2.4. Electrochemical Measurements

The working electrodes were fabricated by homogeneously mixing the active carbon material (80 wt%), conductive carbon black (10 wt%), and polytetrafluoroethylene (PTFE, 10 wt%) binder in a solvent to form a uniform slurry. This slurry was then rolled into a thin, continuous film. Circular electrodes with a diameter of 1.0 cm were punched from this film, achieving a consistent active material mass loading of approximately 5.0 mg cm^−2^. The prepared electrodes were dried under vacuum at 110 °C for 4 h prior to cell assembly to ensure the removal of residual solvent and moisture.

Symmetrical supercapacitor devices were configured in a standard CR2016 coin-cell architecture. Two identical carbon-based electrodes were assembled facing each other, separated by a porous membrane. The choice of membrane was tailored to the electrolyte: a nonwoven fabric (e.g., TF4030) was used with the 6 M KOH aqueous electrolyte, while a cellulose–based membrane was employed for the ionic liquid electrolyte (EMIMBF_4_) to ensure optimal ionic conductivity and chemical compatibility.

The electrochemical performance of the assembled supercapacitors was evaluated using a CHI760E electrochemical workstation (Shanghai Chenhua Instrument Co., Shanghai, China). Cyclic voltammetry (CV) and galvanostatic charge–discharge (GCD) measurements were conducted to assess charge storage behavior and rate capability. The operational voltage window was set from 0 to 1.0 V for the aqueous system (6 M KOH) and from 0 to 3.6 V for the ionic liquid system (EMIMBF_4_), reflecting their respective electrochemical stability limits.

Electrochemical impedance spectroscopy (EIS) was employed to analyze the internal resistance and ion transport kinetics within the devices. These measurements were conducted at the open-circuit potential by applying a sinusoidal signal with an amplitude of 5 mV across a frequency range of 0.01 Hz to 100 kHz.

The specific capacitance (*C*_g_, F g^−1^) of a single electrode was calculated from the discharge branch of the GCD curves using Equation (S1), which accounts for the discharge time, current, active mass, and voltage window. Subsequently, the energy density (E, Wh kg^−1^) and power density (P, W kg^−1^) of the full symmetrical cell were determined using Equations (S2) and (S3), respectively, providing key metrics for evaluating the device-level performance.

## 3. Results and Discussions

**Figure 2:**
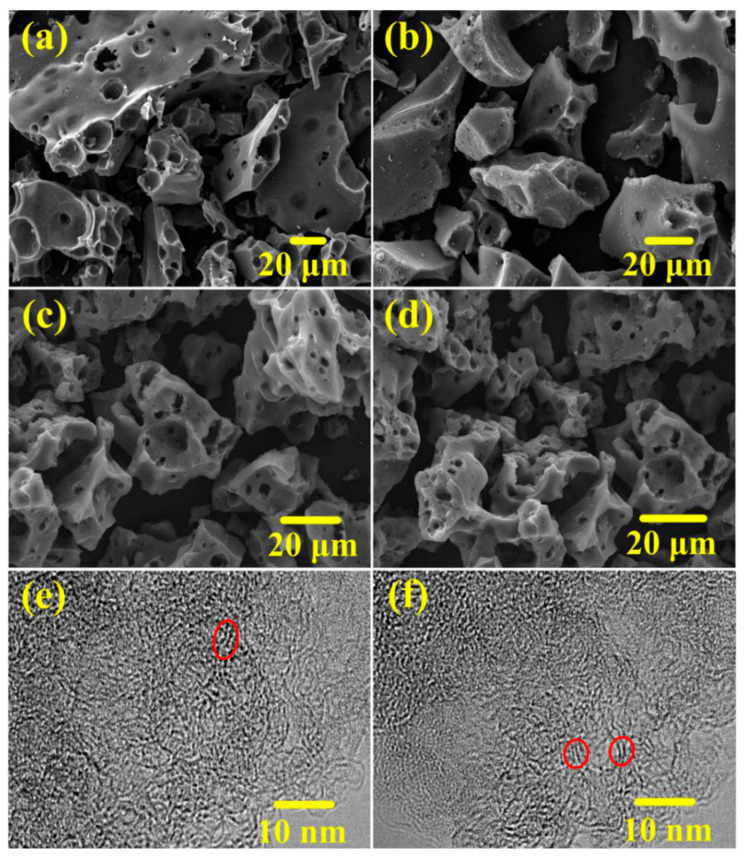
SEM images of (**a**) ALK_0_, (**b**) ALK_0.25_, (**c**) ALK_0.50_ and (**d**) ALK_0.75_, and TEM images of (**e**) ALK_0.50_ and (**f**) ALK_0.75_.

Figure 2 presents SEM and HRTEM images illustrating the morphological and microstructural evolution of the porous carbons (ALK_0_ and ALK_x_ series) as a function of KOH activation intensity. The ALK_0_ sample, synthesized from ammonium lignosulfonate without KOH activation, displays a particulate powder morphology with particle sizes broadly distributed between 20 and 100 μm (Figure 2a). The introduction of KOH activation at a mass ratio (KOH/AL) of 0.25 (sample ALK_0.25_, Figure 2b) leads to a noticeable reduction in particle size compared to ALK_0_. As the KOH/AL mass ratio is progressively increased from 0.25 to 0.50 (Figure 2c) and 0.75 (Figure 2d), the average particle size of the resulting ALK_x_ materials further decreases, while the prevalence of interconnected pores within the carbon structure becomes significantly more pronounced.

HRTEM analysis of samples ALK_0.50_ (Figure 2e) and ALK_0.75_ (Figure 2f) reveals the development of complex nanoporosity, including abundant micropores, alongside the presence of randomly oriented graphitic lattice fringes. These fringes, indicated by red circles in the images, correspond to the (002) diffraction planes of turbostratic carbon [47]. The intensity of the KOH activation directly influences the degree of structural ordering. Specifically, the higher activation ratio used for ALK_0.75_ appears to more effectively etch away disordered carbon phases, resulting in carbon lattice fringes that are more clearly defined and uniform than those observed in ALK_0.50_, suggesting enhanced graphitization at higher KOH/AL ratios [48].

**Figure 3:**
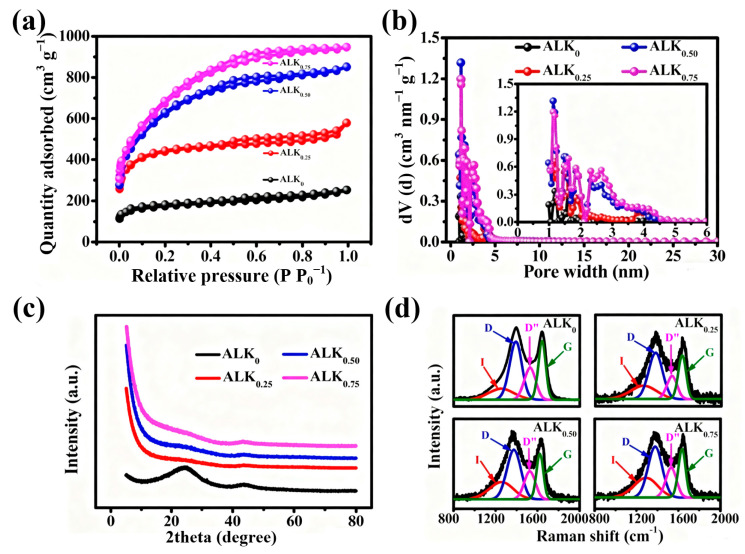
(**a**) N_2_ adsorption–desorption isotherms, (**b**) pore size distribution curves, (**c**) XRD patterns and (**d**) Raman spectra for ALK_0_ and ALK_x_.

The N_2_ adsorption–desorption isotherms of the ALK_0_ and ALK_x_ carbon series, shown in Figure 3a, conform to the Type IV pattern as defined by IUPAC, confirming their mesoporous nature. Significant nitrogen uptake is observed at very low relative pressures (P/P_0_ < 0.05), suggesting the presence of micropores within the carbon matrix. As the relative pressure increases, a prominent hysteresis loop emerges within the P/P_0_ range of 0.4 to 0.9. This hysteresis, coupled with a sharp rise in the adsorbed volume, indicates capillary condensation within a well-developed mesoporous network. The shape and closure point of the hysteresis loop can provide further insight into the pore geometry, such as the presence of slit-shaped or ink-bottle-like pores. The combination of microporosity and extensive mesoporosity in these materials is advantageous for applications requiring rapid ion transport and high surface area for charge storage, as the micropores contribute significantly to the specific surface area while the mesopores facilitate mass transfer [10,49]. The total N_2_ adsorption of ALK_0.75_ is markedly greater than the other samples, suggesting it possesses a substantially larger specific surface area.

The pore size distribution curves derived from the isotherms are shown in Figure 3b. Both ALK_0.50_ and ALK_0.75_ display hierarchical porous structures, featuring a combination of micropores (1–2 nm) and a narrow distribution of small mesopores concentrated in the 2–4.5 nm range. This multi-scale porosity is primarily created through the chemical activation process with KOH. The textural parameters summarized in Table 1 quantitatively support these observations. Compared to ALK_0_, sample ALK_0.25_ shows a noticeable increase in micropore volume due to initial KOH etching. The textural properties of the carbon materials demonstrated a clear dependence on the KOH/AL mass ratio, with systematic enhancement observed as the ratio increased from 0.25 to 0.75. Elevating the KOH/AL ratio to 0.50 significantly promoted the development of mesoporous structure, leading to a notable increase in mesopore volume compared to the ALK_0.25_ sample. This trend culminated in the ALK_0.75_ material, which was synthesized at the highest KOH/AL ratio of 0.75 and exhibited optimal porosity parameters. Specifically, ALK_0.75_ achieved a specific surface area of 2406 m^2^ g^−1^, a total pore volume of 1.47 cm^3^ g^−1^, and a mesopore volume of 0.99 cm^3^ g^−1^, indicating that approximately 67% of the total porosity was contributed by mesopores. The increase in mesopore volume with higher KOH/AL ratios suggests that intensified chemical activation enlarged the pore structure, likely through etching and pore expansion during high–temperature treatment.

The XRD patterns (Figure 3c) show two broad peaks at 23° and 43°, corresponding to the (002) and (100) planes of turbostratic carbon, a disordered graphite-like structure [50]. As the KOH/AL ratio increases, the (002) peak intensity decreases, and the low–angle scattering intensity increases. This indicates the introduction of more nanopores, which increases disorder within the carbon layers [51].

Figure 3d displays the deconvoluted Raman spectra of the ALK_0_ and ALK_x_ carbon materials. The spectra were systematically fitted with four distinct vibrational modes to elucidate their structural characteristics. The spectral decomposition reveals the following features: (1) a broad I–peak centered near 1250 cm^−1^, indicative of heteroatom–induced lattice disorders; (2) a prominent D–peak around 1370 cm^−1^, characteristic of sp^3^–hybridized carbon domains and structural defects; (3) a D′′–peak located at approximately 1530 cm^−1^, often associated with graphitic stacking faults or interfacial distortions; (4) a sharp G–peak near 1580 cm^−1^, corresponding to the in-plane stretching vibrations of sp^2^–bonded carbon atoms in ordered graphitic lattices [52]. The relative intensities and full width at half maximum (FWHM) values of these deconvoluted peaks provide critical insights into the degree of structural order/disorder, defect density, and the sp^2^/sp^3^ carbon hybridization ratio within the materials. The intensity ratio of the D–peak to the G–peak (*I*_D_/*I*_G_) is widely employed to quantify the defect density in carbon-based systems, while the relative prominence of the I-peak can reflect the extent of heteroatom-mediated disorder [53,54,55]. The evolution of these peaks provides detailed insight into the microstructural changes induced by KOH activation.

The XRD analysis reveals a clear correlation between the KOH/AL mass ratio and the structural evolution of the carbon materials. As the ratio increases, the (100) diffraction peak becomes more intense and well–defined. This enhancement indicates improved in-plane structural coherence and lateral expansion of the graphitic domains within the carbon matrix. The sharpening of the peak suggests reduced structural disorder and a transition towards a more ordered crystalline arrangement, likely due to the promoted growth and improved alignment of the graphitic layers during the high–temperature activation process [56]. The Raman data corroborates this trend. The relative area of the I–peak increases with the KOH/AL ratio, indicating a higher concentration of heteroatoms incorporated into the carbon matrix. Concurrently, the D-peak content decreases as the ratio rises from 0.25 to 0.75, reflecting a reduction in sp^3^-defect sites. The more prominent D′′–peak, indicative of turbostratic stacking disorders, is often associated with the creation of interlayer nanopores [57]. The consistent enhancement in the intensity of the G–peak (∼1580 cm^−1^) reflects a gradual expansion of the ordered sp^2^–hybridized carbon network. The *I*_D_/*I*_G_ intensity ratio decreases systematically from 0.51 for ALK_0.25_ to 0.58 for ALK_0.75_ (Table S2).

This inverse relationship between the *I*_D_/*I*_G_ ratio and the degree of graphitization quantitatively confirms a reduction in lattice defects and an improvement in the long-range structural order within the carbon matrix. The observed trend indicates that more extensive chemical activation promotes the formation of a more crystalline graphitic structure, which is advantageous for electrical conductivity and charge transfer kinetics in the electrode material [57].

The surface chemical states and elemental composition of the ALK_x_ samples were analyzed by X-ray photoelectron spectroscopy (XPS), with quantitative results summarized in Table 2. ALK_0.25_ exhibits significantly higher oxygen (7.8 at.%) and sulfur (1.5 at.%) contents compared to ALK_0_, indicating that KOH activation promotes the formation and retention of oxygen- and sulfur-containing functional groups [48,58]. As the KOH/AL mass ratio increases from 0.25 to 0.75, the O and S contents rise progressively, reaching maximum values of 10.0 at.% and 2.5 at.%, respectively, in ALK_0.75_. Conversely, the nitrogen content decreases from 1.9 at.% to 0.7 at.% over the same range, suggesting that nitrogenous groups, which may act as structural templates during carbonization, are progressively eliminated by the intensified KOH etching at higher ratios.

High-resolution C1s spectra (Figure 3a) were deconvoluted into four characteristic peaks, he sp^2^-hybridized C–C bond at approximately 284.8 eV, C–O/C–N at 285.9 eV, C=O/C=N at 286.6 eV, and carboxyl/carbonate groups (COOR) near 289.0 eV [37,59,60,61]. As the KOH/AL ratio increased, the relative area of the C–C peak decreased, while the contributions from C–O/C–N, C=O/C=N, and COOR increased. This is consistent with enhanced incorporation of oxygen and nitrogen heteroatoms into the carbon matrix.

**Figure 4:**
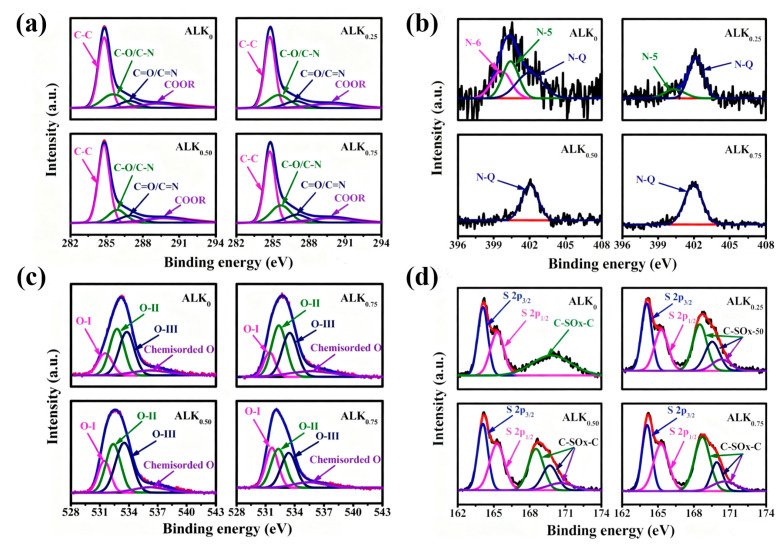
High–resolution XPS spectra of (**a**) C1s, (**b**) N 1s, (**c**) O1s and (**d**) S 2p for ALK_0_, ALK_0.25_, ALK_0.50_ and ALK_0.75_.

The N1s spectra (Figure 4b) revealed three nitrogen configurations: pyridinic N (N–6, ~399.3 eV), pyrrolic N (N–5, ~400.5 eV), and graphitic N (N–Q, ~401.7 eV) [62,63,64]. As the KOH/AL ratio rose, the intensities of the N–6 and N–5 peaks diminished substantially, implying that these less stable nitrogen species were preferentially removed during activation. This process may have concurrently contributed to nanopore development [48]. In contrast, the stable graphitic N (N–Q) species, known to enhance the electrical conductivity of carbon electrodes, became relatively more dominant at higher activation levels.

Deconvolution of the O1s spectra (Figure 4b) identifies four oxygen species: quinone-type C=O (O–I, ~531.5 eV), phenolic/hydroxyl or ether groups (O–II, ~532.3 eV), carboxylic groups (O–III, ~533.5 eV), and chemisorbed oxygen or water (~536.0 eV) [36,65]. The quinone groups (O–I) are particularly significant; they improve electrode wettability, facilitating ion access to the active surface, and can also contribute additional pseudocapacitance in aqueous electrolytes, thereby enhancing the overall charge storage capability [66,67].

High-resolution XPS analysis of the S2p region provides detailed insights into the sulfur chemical states in the carbon materials before and after KOH activation. Figure 4d presents the spectrum for the ALK_0_ ample, which was deconvoluted into three primary components. The doublet with binding energies centered at approximately 164.1 eV (S2p_3/2_) and 165.5 eV (S2p_1/2_) indicates covalent C– bonds, confirming the successful incorporation of sulfur into the carbon matrix. A third component at a higher binding energy of 169.8 eV is attributed to sulfur in an oxidized state, likely in the form of sulfone or sulfate groups [68].

The KOH activation process induces a significant transformation in the sulfur chemistry. The S2p spectra of the ALK_x_ materials reveal the emergence of two distinct new peaks at approximately 168.5 eV and 170.3 eV. The 168.5 eV peak can be assigned to an intermediate oxidation state sulfur moiety, such as a sulfoxide (–C–O–C–) or sulfinic acid (–SO_2_H). The 170.3 eV peak corresponds to a highly oxidized sulfur species, typically identified as a sulfone group (–C–SO_2_–C–) or a sulfate (–SO_3_). The appearance of these peaks suggests that the harsh alkaline activation environment promotes the progressive oxidation of the originally incorporated sulfur, leading to a more complex and diverse surface chemistry that could influence the material’s wettability and electrochemical activity [36].

**Figure 5:**
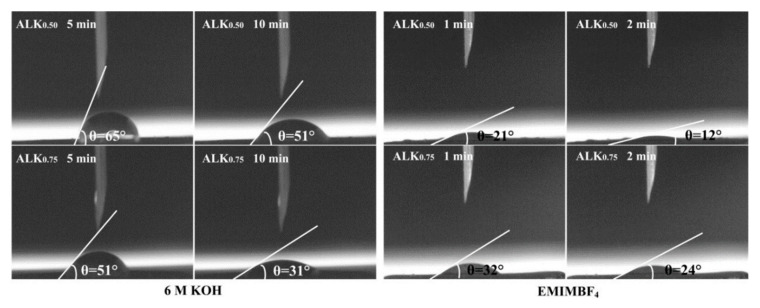
The variation trend of contact angle at different times for ALK_0.50_ and ALK_0.75_.

The strategic incorporation of sulfur and oxygen heteroatoms into the carbon framework induces a synergistic modification of the electrode’s surface chemistry and microstructure. This dual-doping approach introduces polar functional groups (e.g., C–S, C=O, and –OH), which markedly improve interfacial affinity toward aqueous electrolytes. Consequently, the enhanced wettability facilitates efficient ion accessibility and reduces charge-transfer resistance at the electrode–electrolyte interface. However, this effect can be less favorable in non–aqueous electrolytes like ionic liquids, where excessive surface polarity may not be advantageous. As shown in Figure 5, the ALK_0.75_ electrode, benefiting from a higher degree of N, O, S co-doping, demonstrates a lower contact angle in 6 M KOH electrolyte across the entire measured time range compared to ALK_0.50_. This indicates superior surface accessibility and improved electrolyte penetration for ALK_0.75_ in the aqueous system.

Interestingly, despite a similar porous structure to ALK_0.75_, the ALK_0.50_ electrode with lower heteroatom content exhibits a significantly lower contact angle in the EMIMBF_4_ ionic liquid electrolyte over the same testing period. This suggests that a moderately hydrophilic surface, rather than an extensively functionalized one, is more conducive to achieving better wettability with non-aqueous electrolytes.

**Figure 6:**
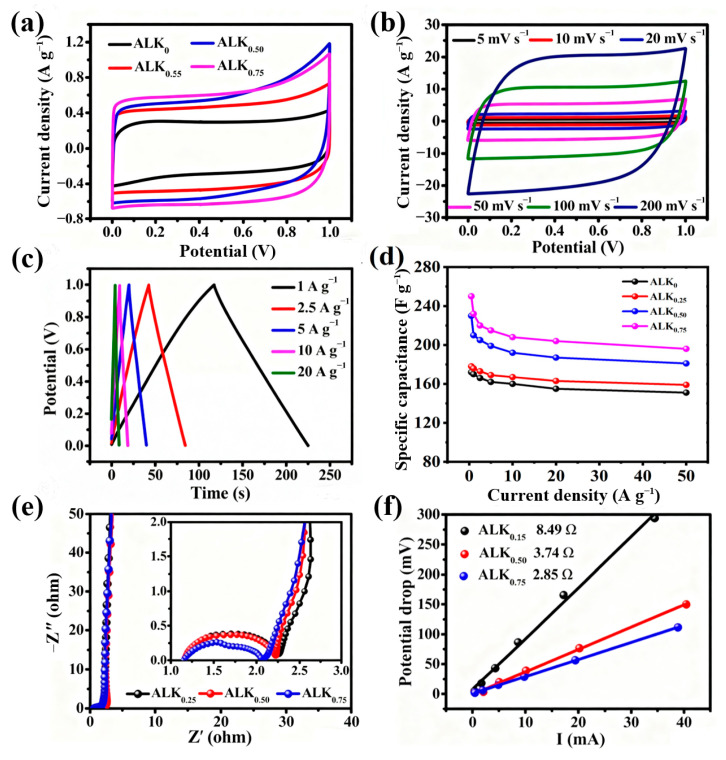
Electrochemical tests of ALK_0_ and ALK_x_ were first carried out in symmetrical cells using 6 M KOH electrolyte: (**a**) the CV curves of four samples at 5 mV s^−1^; (**b**) the CV curves of ALK_0.75_ from 5 to 200 mV s^−1^; (**c**) the GCD curves of ALK_0.75_ at different current densities; (**d**) the specific capacitance of ALK_x_ from 0.25 to 50 A g^−1^; (**e**) Nyquist plots of ALK_x_; (**f**) the relationship between potential drop and current of ALK_x_.

The electrochemical performance of ALK_0_ and ALK_x_ materials was first assessed in a 6 M KOH electrolyte using two-electrode setup. Cyclic voltammetry measurements conducted at 5 mV·s^−1^ (Figure 6a) indicate that ALK_0.75_ yields a markedly greater integrated CV area relative to the other samples, reflecting a superior specific capacitance. This enhancement is likely due to the combined benefits of its increased heteroatom doping level and optimized porous architecture.

The GCD characteristics of the ALK_x_ series were further examined to evaluate their charge storage kinetics and rate performance. As shown in Figure 6c, the GCD curves of all samples, particularly ALK_0.75_, exhibit nearly ideal triangular shapes with highly symmetric charge and discharge branches across varying current densities. This symmetry indicates a highly reversible faradaic process and minimal polarization, resulting in a coulombic efficiency approaching 100%. Figure S1 displays the cycling performance of ALK_0.75_ measured using a two–electrode configuration. After 10,000 cycles, it exhibits an outstanding capacity retention of 98.2%, which convincingly demonstrates its excellent reversibility and validates its potential for application in commercial devices. Figure 6d quantitatively compares the specific capacitance retention of the ALK_x_ electrodes over a wide range of current densities, from 0.25 to 50 A g^−1^. The ALK_0.75_ electrode achieves a maximum specific capacitance of 250 F g^−1^ at the lowest current density of 0.25 A g^−1^, attributable to its optimized heteroatom content and porous structure, which facilitate efficient electrolyte ion accessibility. Due to an ultra–high current density of 50 A g^−1^, it maintains a capacitance of 197 F g^−1^, corresponding to an exceptional capacitance retention of 78.8%. This outstanding rate capability can be ascribed to the synergistic effects of enhanced surface wettability, provided by the polar functional groups from heteroatom doping, and a well-defined pore architecture that promotes rapid ion transport while minimizing diffusion resistance. Such a combination ensures that a high proportion of the electrochemical active sites remains accessible even under extreme charge–discharge conditions. The performance difference between ALK_0.75_ and other samples under high-current operation highlights the critical role of material composition and texture in governing rate performance. The combination of favorable surface chemistry and an interconnected porous network enables ALK_0.75_ to maintain high capacitance and energy efficiency across a wide dynamic range, a crucial requirement for high–power energy storage devices.

The EIS analysis of the ALK_x_ series, as shown in Figure 6e, reveals distinct interfacial charge transfer characteristics and bulk resistive properties. The Nyquist plots exhibit typical features for porous carbon–based electrodes, with medium–frequency semicircular arcs and high–frequency real-axis intercepts providing critical insights into the electrochemical behavior of these materials. The diameter of the medium-frequency semicircular arcs corresponds to the charge transfer resistance (R_ct_), which governs the kinetics of faradaic processes at the electrode-electrolyte interface. The high–frequency intercept on the real axis (Z′) reflects the equivalent series resistance (ESR), encompassing contributions from electrolyte resistance, intrinsic electrode conductivity, and contact resistances [36,69]. Among the synthesized materials, ALK_0.75_ demonstrates superior electrochemical characteristics with the smallest semicircle diameter in the medium–frequency range, indicating significantly reduced charge transfer resistance. This suggests facilitated interfacial ion exchange kinetics, attributable to its optimized heteroatom configuration and surface chemistry. Concurrently, ALK_0.75_ exhibits the shortest real-axis intercept at high frequencies, revealing minimized equivalent series resistance. This combination of low R_ct_ and low ESR underscores the material’s exceptional charge transfer efficiency and rapid ion transport capabilities. The tailored heteroatom doping profile and hierarchical pore structure of ALK_0.75_ contribute to its enhanced electrical conductivity and interfacial properties. These features facilitate efficient charge propagation while minimizing resistive losses, making ALK_0.75_ the most promising candidate for high–performance energy storage applications in the ALK_x_ series.

Figure 6f illustrates the correlation between the applied current and the resulting voltage drop (IR drop) across the ALK_x_ electrodes. A linear increase in IR drop is observed with escalating current, which aligns with the fundamental principle expressed by the equation ΔU = I × R, where ΔU denotes the voltage drop, I represents the current, and R corresponds to the overall internal resistance of the electrochemical system. The slope of the linearly fitted curve quantitatively represents this aggregate internal resistance (R), encompassing contributions from the electrolyte resistance, interfacial charge transfer resistance, and the intrinsic resistance of the electrode materials. The pronounced linearity indicates that the dominant resistance within the device is ohmic in nature. This relationship underscores the critical influence of internal resistance on energy loss during device operation, particularly under high-current conditions. Among the ALK_x_ series, the electrode exhibiting the smallest slope in this plot would indicate the lowest internal resistance, which is a key attribute for minimizing energy loss and enhancing rate capability in practical applications. The internal resistance values for ALK_0.25_, ALK_0.50_, and ALK_0.75_ are determined to be 8.49 Ω, 3.74 Ω, and 2.85 Ω, respectively. This trend of decreasing resistance with increasing KOH/AL ratio is fully consistent with the findings from the EIS analysis, confirming that a higher activation degree leads to enhanced electrode conductivity.

Figure 6f displays the correlation between the applied current and the resulting voltage drop (IR drop) across the ALK_x_ electrodes. The IR drop increases linearly with the current, consistent with the principle expressed by the equation ΔU = I × R, where ΔU is the voltage drop, I is the current, and R is the total internal resistance of the electrochemical system. The slope of the linear fit represents this internal resistance (R), which includes contributions from the electrolyte resistance, interfacial charge transfer resistance, and the intrinsic resistance of the electrode materials. The pronounced linearity suggests that ohmic resistance predominates within the device. This relationship highlights the significant impact of internal resistance on energy loss during device operation, especially at high currents. Within the ALK_x_ series, the electrode with the smallest slope in this plot indicates the lowest internal resistance, a crucial factor for minimizing energy loss and improving rate capability in practical applications. The internal resistance values for ALK_0.25_, ALK_0.50_, and ALK_0.75_ are 8.49 Ω, 3.74 Ω, and 2.85 Ω, respectively. This trend of decreasing resistance with an increasing KOH/AL ratio aligns with the EIS analysis findings, confirming that a higher activation degree enhances electrode conductivity.

**Figure 7:**
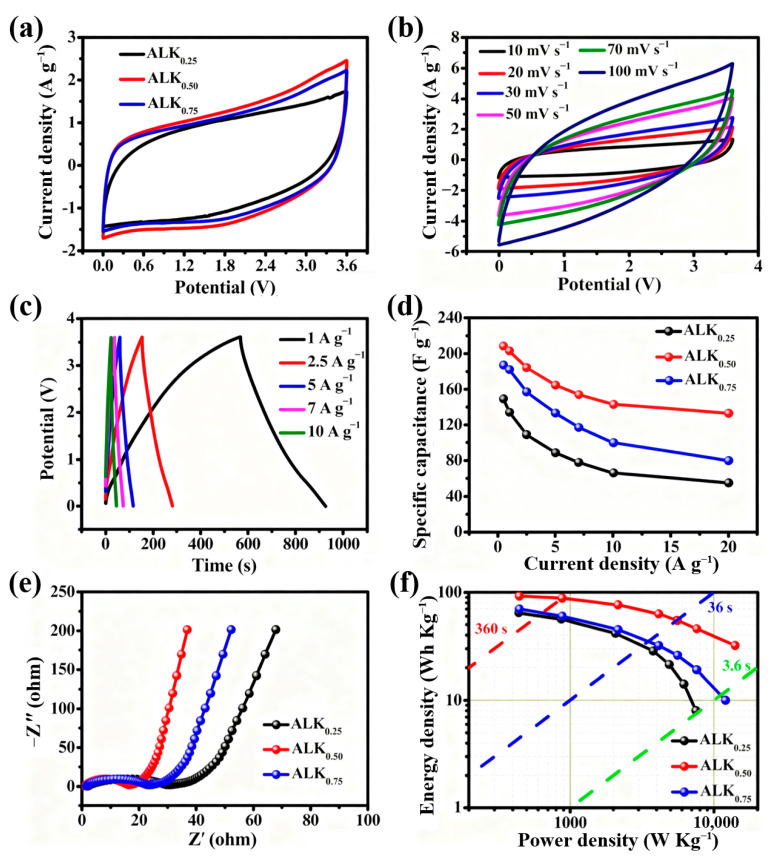
Electrochemical performances of ALK_x_ were further evaluated based on two–electrode supercapacitors in EMIMBF_4_ electrolyte: (**a**) the CV curves of ALK_x_ at 10 mV s^−1^; (**b**) the CV curves of ALK_0.50_ from 10 to 100 mV s^−1^; (**c**) the GCD curves of ALK_0.50_ from 1 to 10 A g^−1^; (**d**) the specific capacitance of ALK_x_ from 0.5 to 20 A g^−1^; (**e**) Nyquist plots of ALK_x_; (**f**) Ragone plots related to energy density and power density of ALK_x_ based symmetric supercapacitors.

The ALK_x_ materials were evaluated in an ionic liquid electrolyte system using EMIMBF_4_ to assess their electrochemical performance under broader operational conditions. As shown in Figure 7a, CV scans at 10 mV·s^−1^ reveal that all ALK_x_ samples exhibit a well-defined quasi-rectangular profile across a voltage range of 0 to 3.6 V. This shape retention indicates highly reversible charge–discharge characteristics and near–ideal EDLC behavior, suggesting compatibility between the electrode materials and the ionic liquid electrolyte. The ability to achieve a stable voltage window of up to 3.6 V, a significant expansion compared to conventional aqueous systems, can be attributed to the intrinsic properties of the EMIMBF_4_ electrolyte. Ionic liquids like EMIMBF_4_ possess a wide electrochemical stability window and low vapor pressure, enabling high–voltage operation without substantial electrolyte decomposition. The minimal distortion observed in the CV curves further suggests rapid ion transport and effective charge propagation within the porous architecture of the ALK_x_ electrodes, highlighting the synergistic effects of material design and electrolyte selection. This combination of ideal capacitive response and an expanded operational voltage window positions the ALK_x_/EMIMBF_4_ system as a promising candidate for high–energy–density supercapacitors, bridging the gap between material properties and electrolyte engineering for advanced energy storage applications. Under these conditions, the ALK_0.50_ electrode demonstrates a higher current density response than ALK_0.75_, indicating its superior charge storage capability in this electrolyte system.

The specific capacitance of porous carbon electrodes in an electrolyte is mainly determined by ion diffusion efficiency, which is influenced by the interplay of pore structure and surface chemistry. Although the pore architecture is largely consistent between ALK_0.50_ and ALK_0.75_, the latter shows a significant increase in oxygen and sulfur–containing functional groups. While these heteroatoms improve surface wettability in aqueous KOH electrolyte, their strong polarity may be less compatible with the non-aqueous EMIMBF_4_ ionic liquid, potentially hindering ion transport. This explains the observed higher capacitance for ALK_0.50_, which has a more moderate surface chemistry profile that is ideal for this electrolyte. The electrochemical characteristics of the ALK_0.50_ electrode were examined under dynamic operating conditions. As shown in Figure 7b, the CV curve recorded at a high scan rate of 100 mV·s^−1^ maintains a quasi-rectangular profile with minimal distortion. This shape retention signifies ideal capacitive behavior and a rapid current response to potential changes, indicating efficient ion transport and easy charge–transfer kinetics at the electrode/electrolyte interface. The low resistance to charge transfer is further demonstrated by the highly symmetrical triangular profiles of the GCD curves across a wide range of current densities (Figure 7c).

The minimal voltage drop (IR drop) and near-perfect coulombic efficiency observed during extended cycling indicate highly reversible faradaic reactions and robust structural stability of the electrode material. ALK_0.50_’s exceptional performance can be attributed to its optimized pore structure and favorable surface chemistry. Its well–defined porous network enables rapid electrolyte ion penetration and reduces the solid–state diffusion length. Additionally, the incorporated heteroatoms enhance interfacial wettability and contribute pseudocapacitive benefits, collectively ensuring excellent rate capability and cycling endurance.

The ALK_0.50_–based symmetric supercapacitor exhibits remarkable electrochemical performance in EMIMBF_4_ electrolyte, marked by low internal resistance and excellent rate capability. As shown in Figure 7d, even at a high current density of 10 A g^−1^, the charge–discharge profiles maintain symmetric triangular shapes with a minimal voltage (IR) drop at the charge–discharge switching point. This minimal polarization indicates a very low ESR, suggesting efficient charge transport and ion diffusion kinetics within the electrode structure. A quantitative analysis of the rate-dependent capacitance further emphasizes the electrode’s robustness. At a current density of 0.5 A g^−1^, the electrode achieves a specific capacitance of 210 F g^−1^. Notably, even as the current density rises significantly to 20 A g^−1^, it retains 135 F g^−1^, corresponding to a capacitance retention rate of 64.3%. This performance highlights the material’s ability to maintain capacitive behavior under extreme current loads, a crucial metric for high–power energy storage devices.

The EIS indicates that the ALK_0.50_ electrode exhibits the lowest R_ct_ in the series (Figure 7e), which enhances its energy storage performance. The device achieves an impressive energy density of 90.2 Wh kg^−1^ at a power density of 885.5 W kg^−1^ with a discharge time of 360 s. Remarkably, it retains an energy density of 34.2 Wh kg^−1^ even at an ultra–high power density of 14.1 kW kg^−1^, demonstrating efficient energy retention under high-demand conditions. These performance metrics exceed those of most previously reported porous carbon–based supercapacitors (see Table S3), establishing ALK_0.50_ as a highly promising electrode material for advanced energy storage applications.

## 4. Conclusions

In summary, N–O–S co-doped hierarchical porous carbons, labeled ALK_0.50_ and ALK_0.75_, were synthesized from ammonium lignosulfonate using a straightforward method with a low KOH/precursor mass ratio of less than 0.8. Once the KOH/AL mass ratio reaches or exceeds 0.5, the activation process effectively induces the formation of small mesopores (2–4.5 nm) within the carbon structure. The ALK_0.75_ material boasts a high heteroatom content (N, O, S) of 13.2 at.%, an ultra-high specific surface area of 2406 m^2^ g^−1^, and a superior mesopore ratio of 67.3%. Evaluated as a supercapacitor electrode in a two-electrode system with a 6 M KOH electrolyte, ALK_0.75_ delivers a high specific capacitance of 250 F g^−1^ at 0.25 A g^−1^ and maintains excellent rate capability, achieving 197 F g^−1^ at 50 A g^−1^. Although ALK_0.50_ has a porosity similar to ALK_0.75_, its lower heteroatom content provides an advantage in non-aqueous electrolytes. The reduced surface polarity of ALK_0.50_ enhances wettability with the ionic liquid EMIMBF_4_, which is crucial for high–voltage operation. Consequently, a symmetric supercapacitor using ALK_0.50_ electrodes achieves outstanding performance metrics, including an energy density of 90.2 Wh kg^−1^ at a power density of 885.5 W kg^−1^, with a discharge time of 360 s. Remarkably, it maintains a high energy density of 34.2 Wh kg^−1^ even at an ultra–high power density of 14.1 kW kg^−1^, highlighting its exceptional charge/discharge kinetics and low internal resistance.

## Figures and Tables

**Table 1:** Porous structure parameters of ALK_0_ and ALK_x_.

Samples	S_BET_^a^ (m^2^ g^−1^)	V_t_^b^ (cm^3^ g^−1^)	V_mic_^c^ (cm^3^ g^−1^)	V_mes_^d^ (cm^3^ g^−1^)	V_mes_ V_t_^−1 e^ (%)
ALK_0_	646	0.39	0.20	0.19	48.7
ALK_0.25_	1625	0.90	0.61	0.29	32.2
ALK_0.50_	2208	1.32	0.56	0.76	57.6
ALK_0.75_	2406	1.47	0.48	0.99	67.3

^a^ Specific surface area calculated by BET method. ^b^ Total pore volume determined by N_2_ adsorption at a relative pressure of 0.99. ^c^ Micropore volume calculated by the V-t method (t-plot method micropore analysis). ^d^ Mesopore volume. ^e^ Mesopore ratio.

**Table 2:** Elemental compositions of ALK_0_ and ALK_x_ determined by XPS.

Samples	Carbon (at.%)					Nitrogen (at.%)				Oxygen (at.%)					Sulfur (at.%)			
	Total C	C-C	C-O/ C-N	C=O/ C=N	COOR	Total N	N-6	N-5	N-Q	Total O	O-I	O-II	O-III	Chemist- ordered-O	Total S	S 2p_3/2_	S 2p_1/2_	C-SOx-C
ALK_0_	90.3	53.2	18.2	6.6	12.3	1.9	0.5	0.6	0.8	6.8	1.2	2.5	2.2	0.9	1.0	0.3	0.3	0.4
ALK_0.25_	89.7	50.7	18.4	8.1	12.5	1.0	0	0.2	0.8	7.8	1.1	2.8	2.9	1.0	1.5	0.4	0.4	0.7
ALK_0.50_	88.5	48.3	18.9	8.6	12.7	0.7	0	0	0.7	8.9	1.8	3.0	3.0	1.1	1.9	0.5	0.5	0.9
ALK_0.75_	86.8	45.1	19.4	9.3	13.0	0.7	0	0	0.7	10.0	2.5	3.2	3.0	1.3	2.5	0.5	0.6	1.4

## Data Availability

The data supporting the findings of this study are available within the article and its Supplementary Materials. Additional raw data are available from the corresponding author upon reasonable request.

## Supplementary Materials

The following supporting information can be downloaded at: https://www.mdpi.com/article/10.3390/nano15211633/s1, Table S1. Composition content (wt%) of AL. Table S2. The content (%) of the peaks in Figure 3d. Figure S1. Cycling stability of ALK_0.75_ at 5 A g^−1^ for 10,000 cycles. Table S3. Power density and energy density of symmetric supercapacitors based on porous carbon.
