# Supplementary material for: N–O–S Co–Doped Hierarchical Porous Carbons Prepared by Mild KOH Activation of Ammonium Lignosulfonate for High–Performance Supercapacitors
Source: Nanomaterials (Basel). 2025 Oct 26;15(21):1633. doi: 10.3390/nano15211633 (PMC12610888; doi:10.3390/nano15211633)
Supplement: Supplementary file 1 [file nanomaterials-15-01633-s001.zip › nanomaterials-3947404-supplementary.pdf]

nanomaterials

Supporting Information

N–O–S Co–Doped Hierarchical Porous Carbons Prepared by Mild KOH Activation of Ammonium Lignosulfonate for High–Performance Supercapacitors

Zhendong Jiang [1], Xiaoxiao Xue [2,*], Yaojie Zhang [3,*], Chuanxiang Zhang [4], Wenshu Li [1], Chaoyi Jia [1] and Junwei Tian [1]

1 Department of Mining Engineering, Shanxi Institute of Technology, Yangquan 045000, China; 18623855902@163.com (Z.J.); 19722743666@163.com (W.L.); 18035324889@163.com (C.J.); 13033479307@163.com (J.T.)
2 Mingde College, Henan University of Technology, Zhengzhou 450001, China
3 College of Chemical Engineering, Ordos Institute of Technology, Ordos 017000, China
4 Ordos Institute of Clean Coal Development and Utilization, Henan Polytechnic University, Ordos 017000, China; zcx223@163.com
* Correspondence: xuexx2010@163.com (X.X.); 13603449015@163.com (Y.Z.)

In symmetrical supercapacitor, the specific capacitance C_g (F g^{-1}) of the electrode was calculated as follows:

$$C_g = \frac{2I\Delta t}{m\Delta V} \quad \text{(S1)}$$

where I, Δt, m and ΔV are the discharge current (A), discharge time (s), mass (g) of the carbon material on single electrode and voltage change (V), respectively.

The energy density E (Wh kg^{-1}) and power density P (W kg^{-1}) of symmetrical supercapacitors calculated based on discharge curves are as follows:

$$E = \frac{C_g \Delta V^2}{8\times 3.6} \quad \text{(S2)}$$

$$P = 3600\times\frac{E}{\Delta t} \quad \text{(S3)}$$

where C_g, ΔV, and Δt are the specific capacitance (F g^{-1}) of the single electrode, voltage change (V) and discharge time (s), respectively.

Table S1. Composition content (wt%) of AL.

Samples	Organics	Humic acid	Total Nitrogen	Total Potassium	Sodium
AL	>80	50.8	6.05	5.3	2.23

Table S2. The content (%) of the peaks in Figure 3d.

Samples	I	D	D″	G	I_G/I_D
ALK_0	22.9	37.0	18.3	21.8	0.59
$ALK_{0.25}$	23.4	40.0	16.1	20.5	0.51
$ALK_{0.50}$	24.0	37.3	17.8	20.9	0.56
$ALK_{0.75}$	24.5	36.2	18.1	21.2	0.58

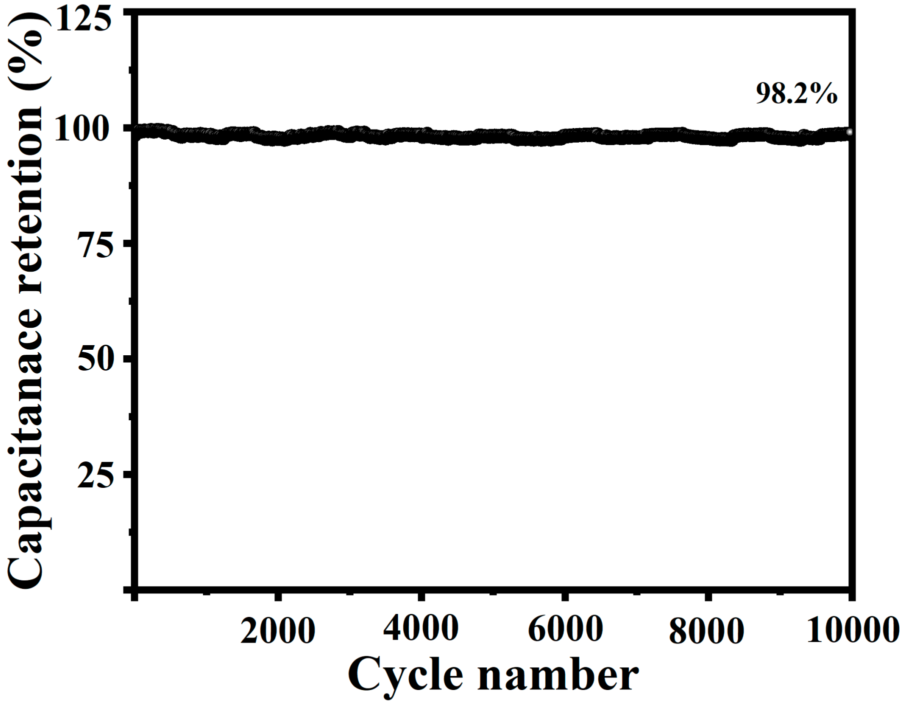

Figure S1. Cycling stability of $ALK_{0.75}$ at 5 A g^{-1} for 10000 cycles.

Table S3. Power density and energy density of symmetric supercapacitors based on porous carbon.

Materials	Specific surface area (m^2 g^{-1})	Electrolyte	Specific power (W kg^{-1})	specific energy (Wh kg^{-1})	Ref.
BAPC-800	3506	$EMIMBF_4$	437.5	82.5	[1]
PNPCN-1.5	2920	$EMIMBF_4$	4.7k	56.29	[2]
HPCS-900	1939	1 M $SBPBF_4$/PC	–	34.3	[3]
			9.4 k	20.5	
PCNS-6	1947	$EMIMBF_4$	375	54.1	[4]
			15 k	25.4	
YP-50F	1364.72	$EMIMBF_4$	496.6	34.8	[5]
N-CNF-750	1003.6	$EMIMBF_4$	1750	60.4	[6]
HPC	1963	7 M Li-TFSI	575	32.9	[7]

			11.5 k	16.3	
BQ/PD-C	2334	LiTFSI	–	31.3	[8]
		$TEMABF_4$/PC	–	60.2	
		$EMIMBF_4$	–	90.6	
NOPC	2900	$EMIMBF_4$	337.6	56.4	[9]
$ALK_{0.50}$	2406	$EMIMBF_4$	885.5	90.2	This work
			14.1 k	34.2	

References

1. Sun, Y. K.; Xu, D.; He, Z. J.; Zhang, Z. H.; Fan, L. W.; Wang, S. R., Green fabrication of pore-modulated carbon aerogels using a biological template for high-energy density supercapacitors. *J. Mater. Chem. A* **2023,** 11, (37), 20011–20020.
2. Sun, Y. K.; He, Z. J.; Fan, H. Y.; Wang, S. R., Nitrogen configuration modulation of porous graphitic carbon nanosheets for superior capacitive energy storage. *J. Power Sources* **2024,** 614, 235027.
3. Pang, J.; Zhang, W. F.; Zhang, H.; Zhang, J. L.; Zhang, H. M.; Cao, G. P.; Han, M. F.; Yang, Y. S., Sustainable nitrogen-containing hierarchical porous carbon spheres derived from sodium lignosulfonate for high-performance supercapacitors. *Carbon* **2018,** 132, 280–293.
4. Chen, C.; Yu, D. F.; Zhao, G. Y.; Du, B. S.; Tang, W.; Sun, L.; Sun, Y.; Besenbacher, F.; Yu, M., Three-dimensional scaffolding framework of porous carbon nanosheets derived from plant wastes for high-performance supercapacitors. *Nano Energy* **2016,** 27, 377–389.
5. Li, Z. M.; Xiao, D. W.; Li, Z. H.; Xu, Z. M.; Dou, H.; Zhang, X. G., Optimizing EMIMBF4-based electrolyte with LiBr redox medium for enhanced supercapacitors. *J. Energy Storage* **2024,** 89, 111735.
6. An, Y. F.; Yang, Y. Y.; Hu, Z. G.; Guo, B. S.; Wang, X. T.; Yang, X.; Zhang, Q. C.; Wu, H. Y., High-performance symmetric supercapacitors based on carbon nanosheets framework with graphene hydrogel architecture derived from cellulose acetate. *J. Power Sources* **2017,** 337, 45–53.
7. Qian, X.; Miao, L.; Jiang, J.; Ping, G.; Xiong, W.; Lv, Y.; Liu, Y.; Gan, L.; Zhu, D.; Liu, M., Hydrangea-like N/O codoped porous carbons for high-energy supercapacitors. *Chem. Eng. J.* **2020,** 388, 124208.
8. A universal strategy to obtain highly redox-active porous carbons for efficient energy storage. *J. Mater. Chem. A* **2020,** 8, 3717–3725.
9. Mao, Y.Q.; Dong, G.H.; Li, Y.Q.; Huang, P.; Fu, S.Y., One-step fabrication of N/O self-doped porous carbon derived from 2-MeIM for high-performance supercapacitor electrode. *J. Energy Storage* **2023,** 74, 109263.
